# ANGPLT3: A novel modulator of lipid metabolism

**DOI:** 10.21542/gcsp.2017.6

**Published:** 2017-03-31

**Authors:** Mohamed Hassan

**Affiliations:** Division of Cardiology, Aswan Heart Centre, Aswan, Egypt

## Abstract

Angiopoietin-like proteins (ANGPTLs) have emerged as an important regulator of lipid and glucose metabolism as well as insulin sensitivity. ANGPTL3 plays a key role in regulating circulating triglycerides (TG) and cholesterol levels through reversible inhibition of lipoprotein lipase (LPL) and endothelial lipase enzymes activity. Loss of function mutation of ANGPTL3 gene has been identified in many subjects with familial combined hypolipidemia. ANGPTL4 produces irreversible inhibition of LPL activity, while ANGPTL8 enhances the activity of ANGPTL3, which highlight the interplay between the different ANGPTLs in a coordinated manner to regulate lipid metabolism during different nutritional states. This new class of lipid modulators may serve as potential novel therapeutic target for reducing plasma lipoprotein and treatment of metabolic syndrome.

## Introduction

Angiopoietin-like proteins (ANGPTLs) are a group of eight proteins that share structural similarity to the angiopoietin protein “vascular endothelial growth factors” family.^1^ ANGPTLs do not bind to the angiopoietin receptor Tie2 or the related protein Tie1, which suggests different functional properties for this family. Growing evidence has demonstrated a central role for these proteins in regulation of lipid and glucose metabolism during different nutritional states. In this review, we will focus on the role of different ANGPTLs - especially ANGPTL3 - in lipid homeostasis, and their proposed link to certain inherited lipoprotein disorders, as well as the potential use of these proteins as a novel therapeutic target for reducing plasma lipoprotein levels.

### ANGPTL3

**Figure 1.**
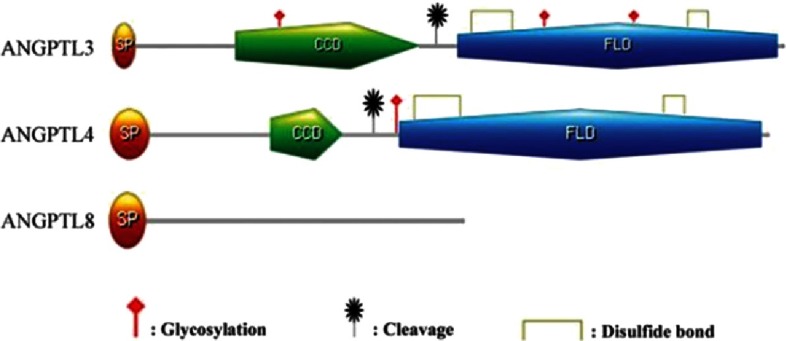
Structural comparison of ANGPTL3, ANGPTL4 and ANGPTL8. Unlike ANGPTL3 and ANGPTL4, ANGPTL8 does not contain glycosylation site, disulfide bond, CCD, or FLD and does not require cleavage to become active (adapted from [1]).

Angiopoietin-like protein 3 (ANGPTL3), also known as angiopoietin-5, is a 460-amino acid glycoprotein secreted almost exclusively from the liver.^1,2^ Human ANGPTL3 contains an N-terminal heparin-binding motif, a coiled-coil domain (CCD), and a C-terminal fibrinogen-like domain (FLD) that binds integrin v3 (Figure 1). It is encoded by a gene located at chromosome 1 p31.1-p22.3, which has seven exons.^1^ *In vivo*, it is proteolytically cleaved by hepatic proprotein convertase between the CCD and the fibrinogen-like domain, and then the full length and cleaved ANGPTL3 circulate in plasma.^3,4^ The N-terminal fragment plays a pivotal role in lipid trafficking and metabolism, while the C-terminal fragment is involved in angiogenesis. ANGPTL3 has emerged as an important regulator of circulating triglycerides (TG) and cholesterol levels through reversible inhibition of lipoprotein lipase (LPL) and endothelial lipase (EL) enzymes activity.^5^

LPL is an enzyme anchored to the capillary endothelium via glycosylphosphatidylinositol- anchored high density lipoprotein-binding protein 1 (GPIHBP1), and is responsible for clearance of circulating of TG rich lipoproteins – chylomicrons and very low density lipoproteins (VLDL) – resulting in the formation of chylomicron remnants and intermediate density lipoproteins (the source of low density lipoprotein “LDL”) respectively.^6^ Free fatty acids (FFAs) liberated during hydrolysis serve as energy source in the muscles, stored as TG in adipose tissue (AT), or repackaged in VLDL in the liver. Cumulative evidence in the last decade revealed that LPL activity is controlled not only by ANGPTL3, but also by another two proteins belonging to the ANGPTL family; ANGPTL4, and ANGPTL8, in addition to apoprotein (APO) C3 and APOC2.^5,7,8^ While ANGPTL3 is mainly active in the feeding state, ANGPTL4 regulates LPL activity during different physiological situations, including fasting.^1,9^

**Figure 2.**
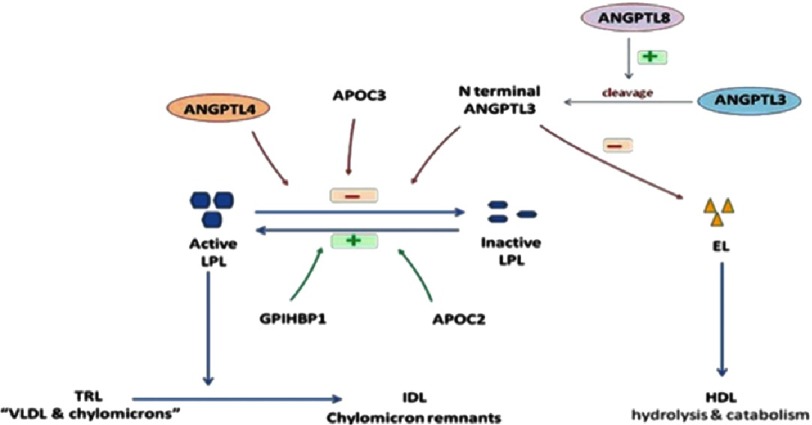
The role of ANGPTLs in lipid metabolism. ANGTPL3, angiopoietin like 3 protein; ANGPTL4: angiopoietin like 4 protein; ANGPTL8, angiopietin like 8 protein; LPL, lipoprotein lipase; EL, endothelial lipase; APOC3, apoprotein C3; APOC2, apoprotein C2; GPIHBP1, glycosylphosphatidylinositol- anchored high density lipoprotein-binding protein 1; TRL, triglyceride rich lipoproteins; VLDL, very low density lipoprotein; HDL, High density lipoprotein.

EL is a member of the TG lipase gene family which is highly homologous to LPL (44%) and hepatic lipase (41%).^10^ EL is an important enzyme in the physiological regulation of high-density lipoproteins (HDL) metabolism.^10^ It is termed endothelial due to the fact that it is synthesized in the endothelial cells. EL hydrolyzes HDL more efficiently than other lipoproteins, and more effectively on HDL particles that contain only APOA-I than on HDL particles that contain both APOA-I and APOA-II (Figure 2).^11,12^

Therefore, ANGPTL3 deficiency results in increased LPL and EL activities, and hence decreased levels of VLDL-C, LDL-C and HDL-C.^13^ Koster et al. have demonstrated 9-fold higher post heparin plasma LPL activity in male ANGPTL3-deficient mice, compared with male wild-type mice.^14^ Subsequently, homozygote carriers of LOF mutation in ANGPTL3 gene showed a marked increase in post heparin plasma LPL activity compared to non-carriers.^15^ ANGPTL3 reduces the availability of circulating FFAs as a consequence of inhibition of lipolysis in AT,^16^ providing lesser substrate for the assembly of nascent VLDL particles.^15,17^ Interestingly, Plasma FFAs, insulin, glucose, and homeostatic model assessment of insulin resistance were found to be significantly lower in homozygous carriers of S17X loss of function (LOF) mutation of ANGPTL3 compared with heterozygotes and non-carriers subjects.^15^ This may be attributed to a reduced FFA level which may improve tissue insulin function.^18^ Therefore, ANGPTL3 may play a pivotal role in modulating not only lipid metabolism, but also glucose metabolism and insulin sensitivity which may highlight the role of ANGPTL3 as a hepatokine that modulates the crosstalk between liver and AT.^15^

Several inherited lipoprotein disorders have been recently linked to ANGPTL3. LOF mutations in ANGPTL3 gene have been demonstrated in patients with familial hypobetalipoproteinemia and familial combined hypolipidemia.^19–21^

### ANGPTL3 and familial hypobetalipoproteinemia

Familial hypobetalipoproteinemia (FHBL) is an autosomal dominant lipoprotein disorder defined as <5th percentile LDL-C or APOB in the plasma.^22^ Subjects are usually heterozygous and asymptomatic with low risk for coronary artery disease (CAD). Approximately 50% of cases are carriers of mutations in the APOB gene that results in truncated APOB with reduced capacity to bind lipids and to form lipoproteins, and hence defective hepatic VLDL export and liver steatosis.^22,23^ Patients may also have fat malabsorption due to defective chylomicron secretion in the intestine. The clinical phenotype of heterozygous FHBL is usually mild, characterized only by fatty liver, however patients with homozygous or compound heterozygous APOB mutations may have severe biochemical and clinical phenotype, similar to the abetalipoproteinemia (caused by mutations in microsomal TG transfer protein “MTP” gene), characterized by intestinal malabsorption, pigmentary retinal degeneration, ataxic neuropathy, and almost undetectable levels of LDL-C and APOB.^20^

The genetic basis of the non-APOB FHBL remained a question for long time. In some kindred, FHBL was linked to a locus on chromosome 3 (3p21) but the candidate gene was unknown.^22^ Subsequently, a FHBL- like phenotype was observed in carriers of LOF mutation of the proprotein convertase subtilisin Kexin-9 (PCSK9) gene that was not associated with fatty liver - as shown in FHBL due to APOB gene mutations.^24,25^ However, no mutations in either APOB or PCSK9 were detected in a large proportion of FHBL subjects.

LOF mutation of ANGPTL3 gene was firstly demonstrated in a strain of obese mice (KK/San strain) with severe recessive hypolipidemia.^26^ Furthermore, treatment with recombinant ANGPTL3 or adenovirus-mediated overproduction of ANGPTL3 has been found to elevate plasma TG and total cholesterol in mice.^27^ Subsequently, LOF alleles of ANGPTL3 were found in the lowest quartile of plasma TG in participants in the Dallas Heart Study.^19^

### ANGPTL3 and familial combined hypolipidemia

Familial combined hypolipidemia is a recently discovered autosomal recessive dyslipidemic phenotype characterized by global reduction of plasma APOB and APOA1 lipoproteins, resulting in very low serum levels of TG, LDL-C, and HDL-C.^21^ There is no evidence that it is associated with liver abnormalities or adverse clinical sequale.^21^ The genetic cause of familial combined hypolipidemia has been attributed to mutations in the ANGPTL3 gene, however the mode of inheritance and clinical implications are not well defined.^21,28^ Exome sequencing of two family members with combined hypolipidemia revealed two distinct nonsense mutations (S17X and E129X) in the ANGPTL3 gene.^28^ Musunuru et al. reported that heterozygous carriers of either S17X or E129X mutations had plasma LDL-C and TG levels that were intermediate between the levels in persons with neither mutation and those with both mutations, findings consistent with a codominant mode of inheritance.^28^

Nonsense and/or missense mutations in ANGPTL3 gene were also demonstrated in 8 subjects (10.25%) - among 78 American and Italian subjects - with combined hypolipidemia. Compound heterozygotes for ANGPTL3 mutations (S17X and E129X) had significantly lower plasma LDL-C, TG, and HDL-C levels than heterozygous carriers of both mutations.^20^ No mutations of APOB, PCSK9, or MTP genes were detected in any subject.^20^ A 15.2 % prevalence of ANGPTL3 mutations has been also reported in another study conducted in Campodimele, a small town located in the province of Latina, Italy.^21^ In contrast with Musunuru et al.,^8^ this study reported that only individuals with two nonsense S17X alleles and no detectable ANGPTL3 in the serum had a significant reduction of LDL-C, TG, and HDL- C levels, a lipid phenotype compatible with familial combined hypolipidemia,^21^ while heterozygous carriers showed a significant reduction of only total cholesterol and HDL-C.^21^

### ANGPTL4

ANGPTL4 is a 460-amino acid glycoprotein, secreted predominantly from the liver and AT. It is encoded by a gene that has seven exons and located at chromosome 19p13.3.^1^ Like ANGPTL3, it is cleaved *in vivo* to generate N- and C-terminal fragments with highly divergent biological activities. ANGPTL4 has emerged as a key determinant of the physiological fluctuations in LPL activity.^9^ During fasting, ANGPTL4 expression increases and promotes irreversible conversion of active LPL dimer into inactive monomers which inhibit the LPL activity and thereby decrease the release of FFA from circulating TG- rich lipoproteins.^29^ After feeding, the ANGPTL4 expression level is markedly decreased, relieving the inhibition of intravascular lipolysis and promoting the uptake of dietary lipids into the AT.

ANGPTL4 is expressed under sensitive transcriptional control of FA and Peroxisome proliferator-activated receptor (PPAR) which is part of a feedback mechanism to protect cells against lipotoxicity.^5^ Therefore plasma ANGPTL4 levels correlate strongly with FFA levels.^15^ On the other hand, recent findings have revealed a critical role for ANGPTL4 in cancer growth and progression, promoting the metastatic processes of melanoma cells and breast tumour cells.^30^

ANGPTL4 overexpression has been reported to increase plasma TG level.^31^ Furthermore, ANGPTL4-deficient mice displayed hypotriglyceridemia and elevated post heparin LPL activity with greater effects in the fasted compared with the fed state.^32^ However, ANGPTL3-deficient mice displayed hypotriglyceridemia with a greater effect in the fed state. Mice deficient in both ANGPTL proteins showed a synergistic effect on plasma TG and did not survive past 2 months of age.^32^ Interestingly, plasma levels of ANGPTL4 were found to be significantly reduced in homozygotes carriers of S17X loss of function mutation of ANGPTL3.^15^ These findings might suggest a coordinate regulation of both ANGPTL3, ANGPTL4 by FFA.

### ANGPTL8

ANGPTL8 (also called betatrophin, or lipasin) is a 198-amino acid secreted protein, encoded by a gene located at chromosome 19p13.2. In mice, ANGPTL8 is abundantly expressed in the liver and AT, however human ANGPTL8 is expressed in the liver only.^1,31,33^

ANGPTL8 shares homology with the N-terminal domain of ANGPTL3 that is released after cleavage and involved in regulation of lipid metabolism.^31^ ANGPTL8 has been identified as an important factor in regulating serum TG levels, and in replenishing the TG store in AT.^1,31,33^ ANGPTL8 promotes cleavage of ANGPTL3 to augment its activity. In addition, it promotes proliferation of pancreatic β-cells, and hence insulin secretion.^33^

ANGPTL8 is nutritionally regulated, as its mRNA levels in liver and fat as well as its protein level in serum are reduced by fasting and upregulated by feeding.^7,34^ ANGPTL8-deficient mice have lower serum TG levels, while adenovirus-mediated overexpression of ANGPTL8 has been reported to increase serum TG levels.^34^ Interestingly, Irisin - a newly identified hormone secreted by the skeletal muscles which promotes ‘browning’ of white AT - promotes the expression of ANGPTL8 and pancreatic beta cell proliferation, and improves glucose tolerance.^35^

## Conclusion

Angiopoietin-like 3 proteins have emerged as a new class of lipid metabolism modulators which may serve as a potential novel therapeutic target for reducing plasma lipoprotein and treatment of metabolic syndrome. These recent advancements are further proof of the clinical usefulness of exome sequencing for identification of novel genetic causes of inherited lipoprotein disorders and uncover novel targets for lipid-lowering therapy.
